# Electron tomography and fractal aspects of MoS_2_ and MoS_2_/Co spheres

**DOI:** 10.1038/s41598-017-12029-8

**Published:** 2017-09-26

**Authors:** Manuel Ramos, Félix Galindo-Hernández, Ilke Arslan, Toby Sanders, José Manuel Domínguez

**Affiliations:** 1 Instituto Mexicano del Petróleo (IMP), Eje Central Lázaro Cárdenas Norte 152 Col. San Bartolo Atepehuacan, México, D.F. C.P 07730 USA; 2 Departamento de Física y Matemáticas, UACJ-Instituto de Ingeniería y Tecnología, #450 Avenida del Charro, Cuidad Juárez, 32310 México, USA; 3 Fundamental and Computational Sciences Directorate, Institute for Integrated Catalysis and Environmental Molecular Science Laboratory, Pacific Northwest National Laboratory, Post Office Box 999, Richland, Washington 99352 United States

## Abstract

A study was made by a combination of 3D electron tomography reconstruction methods and N_2_ adsorption for determining the fractal dimension for nanometric MoS_2_ and MoS_2_/Co catalyst particles. *DFT* methods including Neimarke-Kiselev’s method allowed to determine the particle porosity and fractal arrays at the atomic scale for the S-Mo-S(Co) 2D- layers that conform the spherically shaped catalyst particles. A structural and textural correlation was sought by further characterization performed by x-ray Rietveld refinement and Radial Distribution Function (RDF) methods, electron density maps, computational density functional theory methods and nitrogen adsorption methods altogether, for studying the structural and textural features of spherical MoS_2_ and MoS_2_/Co particles. Neimark-Kiselev’s equations afforded the evaluation of a pore volume variation from 10 to 110 cm^3^/g by cobalt insertion in the MoS_2_ crystallographic lattice, which induces the formation of cavities and throats in between of less than 29 nm, with a curvature radius *r*_*k*_ < 14.4 nm; typical large needle-like arrays having 20 2D layers units correspond to a model consisting of smooth surfaces within these cavities. Decreasing *D*_*P*_, *D*_*B*_, *D*_*I*_ and *D*_*M*_ values occur when Co atoms are present in the MoS_2_ laminates, which promote the formation of smoother edges and denser surfaces that have an influence on the catalytic properties of the S-Mo-S(Co) system.

## Introduction

Catalytic materials are the main pillars of liquid fuels production from hydrocarbons and its role is vital for supplying energy needs from petroleum natural resources. The layered transition metal sulfides (LTMS) such as molybdenum di-sulfide (MoS_2_) were used extensively in the petroleum refining industry for more than five decades and these catalysts are referred by the catalysis research community as the “workhorses” of refining processes^1^. From the industrial viewpoint cobalt and nickel are used as promoters of MoS_2_ catalytic materials for enhancing deep hydrodesulfurization (HDS)^2–7^ for clean fuels production containing ultra-low amounts of sulfur; however, from a basic viewpoint there is no a single model for explaining the promoter effects of Co and Ni. Previous research work by A.L. Farragher and P. Cossee^8^ proposed a model that explains the promoter effect of Co and Ni atoms in terms of a “pseudo-intercalation” effect, where Co atoms are located at octahedral sites of MoS_2_ layered phase edges, i.e., (1010) type planes; also, B. Delmon *et al*. proposed a model based on the formation of a mix phase of MoS_2_ and Co_9_S_8_, which could be formed by synergic interactions in the unsupported systems^9^; H. Topsøe *et al*. used Mössbauer spectroscopy for demonstrating that chemical bonding occurs at the edge planes of Cobalt-Sulfur-Molybdenum, thus coining the term “CoMoS” phase^10^; also, Chianelli *et al*. proposed a “Rim-Edge” model in an attempt for defining the seat of catalytic activity and specific sites within the MoS_2_ stacking structure, which correlates with the activity and selectivity of unsupported systems^11^. More recently, Lauritsen *et al*. reported a combined study using scanning tunneling microscopy (STM) and computer assisted numerical simulations involving plane wave density functional theory methods^12,13^, which led them to conclude that metallic states in the cobalt-sulfur bonding play a key role^14^. Furthermore, MoS_2_ layered packing structures produce diverse morphologies like nanotubes, needles, fullerenes and spheres, as described by Remškar *et al*.^15^, Camacho-Bragado *et al*.^16^, Blanco *et al*.^17^ and Ramos *et al*.^18^. The latter author used TEM and DFT simulations^19^ to show that electron donation of cobalt promoters occurs by synergic contact between Co_9_S_8_ and MoS_2_ phases. Furthermore, non-conventional TEM sample holders allowed *in-situ* and *operando* experiments with these phases, as reported by Ramos *et al*., Helveg *et al*. and Casillas *et al*. These authors showed carburization effects on fresh MoS_2_ and MoS_2_/Co catalysts^20^, crystal formation from molybdenum oxide precursors in the presence of H_2_S and H_2_ as well as variations of micromechanical properties^21,22^. In other works, STEM 3D tomography was used, as reported by P.A Midgley and R.E. Dunin-Borkowski, to characterize low dimensional materials, proteins and organic specimens^23^, while D.A. Muller resolved the atomic structure and chemical bonding of some crystallites^24^. Ziese *et al*. implemented STEM-electron tomography techniques to determine the gold nanoparticles distribution over a SBA-15 type matrix^25^, while Arslan *et al*. reported the use of this technique for locating gold particles supported over alumina oxide matrix^26^. The present work afforded a multi-technique analysis of MoS_2_ and Cobalt-promoted MoS_2_ catalytic particles and a calculation was made of the surface fractal dimension at the molecular level for assessing better the textural and structural features promoted by Co insertion into the MoS_2_ lattice. Surface fractal dimensionality is related to the roughness, steps and kinks at the molecular scale and it plays a key role for heterogeneous catalysts, as pointed out by several authors^27–30^. We propose this parameter as an important one for gauging catalytic behavior of Co-promoted-MoS_2_ catalysts and as a complement of “electronic effects” induced by insertion of the Co promoter, because it modifies both textural and structural properties of MoS_2_; this is the first time that fractal dimensionality is shown as a result of Co-promoter insertion in spherical particles of MoS_2_ and the profound effects on both texture and structure as verified in the case of MoS_2_/Co system.

## Results and Discussion

**Figure 1:**
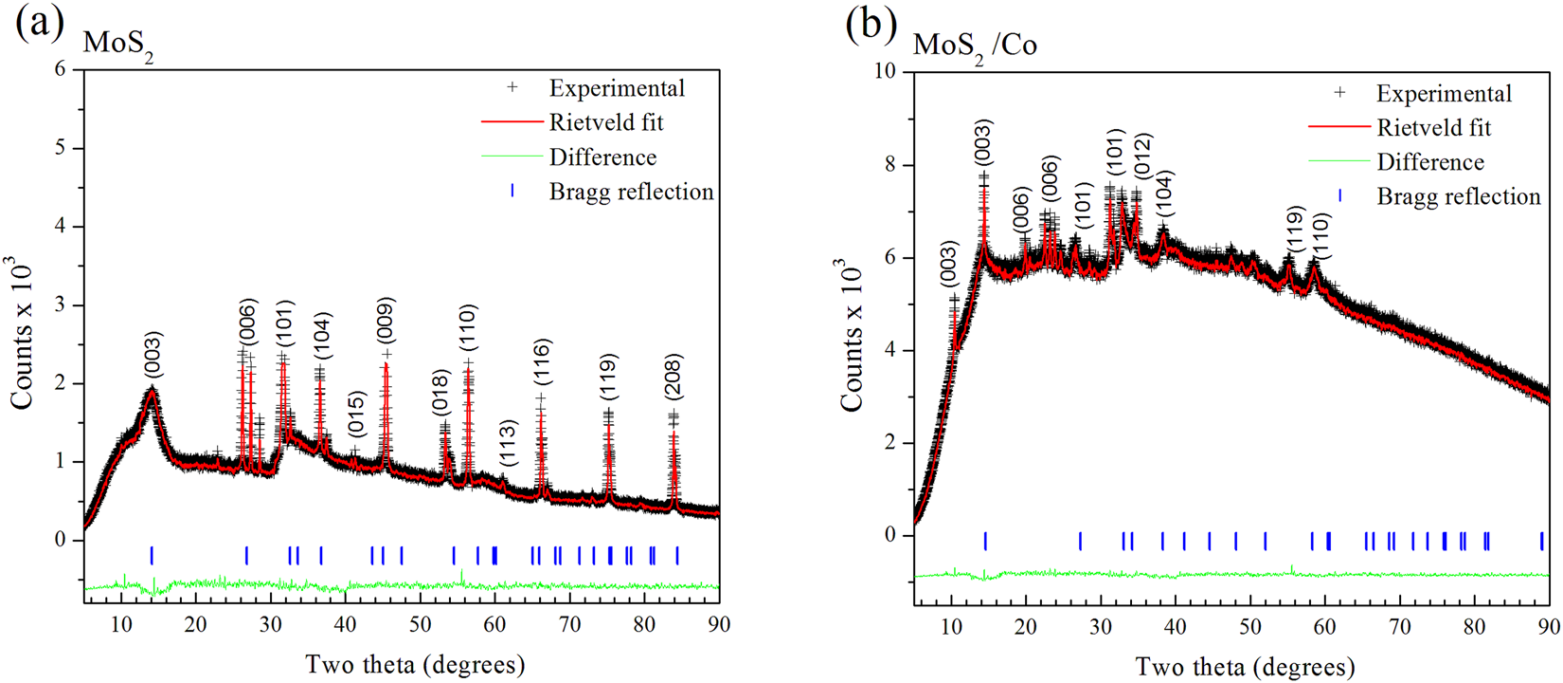
Powder x-ray diffraction patterns and Rietveld refinement for (**a**) MoS_2_ and (**b**) Co/MoS_2_ samples, it is possible to observe a sharp peak at (003)-basal plane when cobalt atoms are present, compared to pure MoS_2_ .

**Figure 2:**
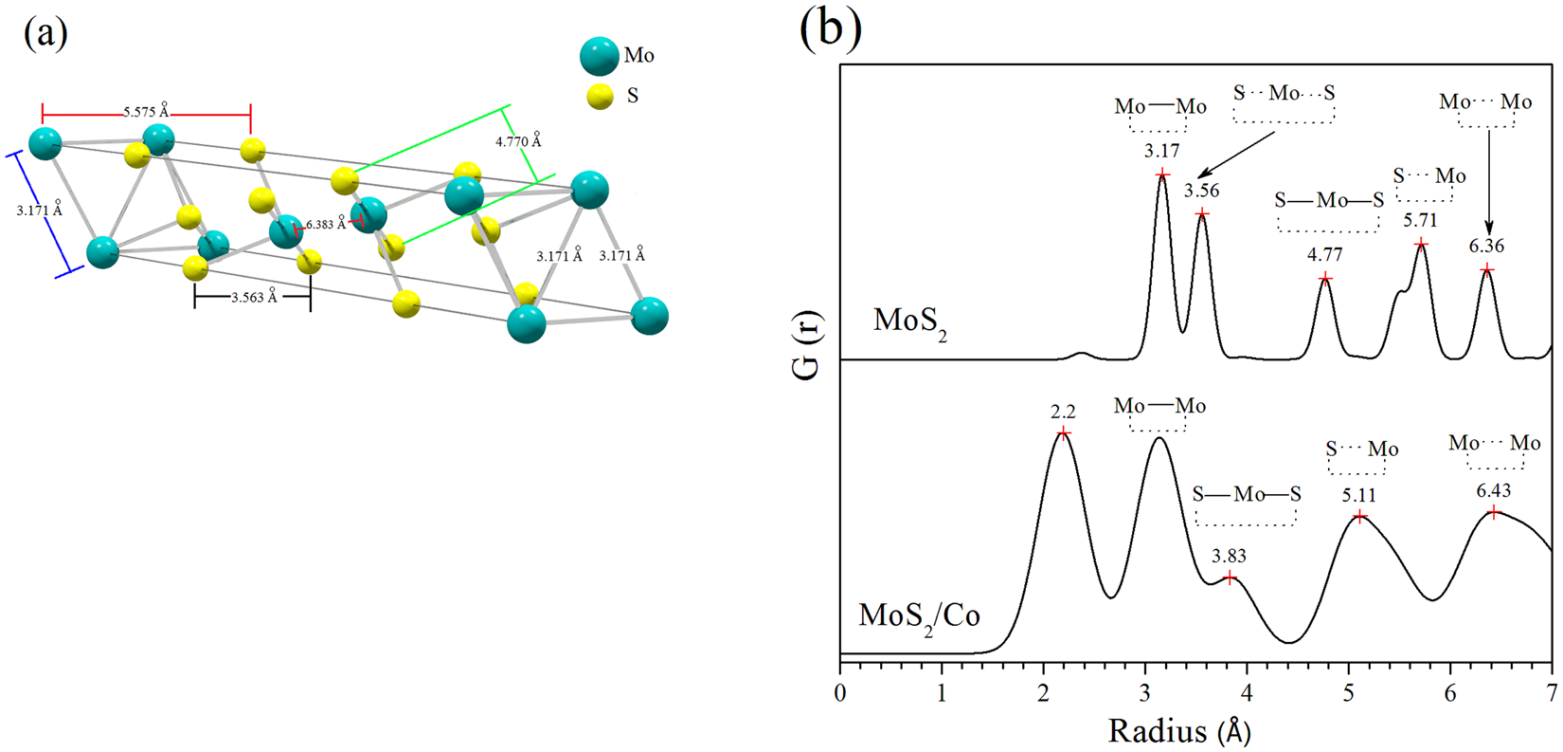
Radial distribution plots for (**a**) MoS_2_ crystallographic lattice (a = b = 3.1710 and c = 18.3445 Å) (SG: R3mH). (**b**) Co/MoS_2_ it is possible to observe a shifting of Mo-Mo and S-Mo-S peaks, as well the appearance of a new peak at 2.2 Å when cobalt atoms are present in the MoS_2_ lattice.

**Figure 3:**
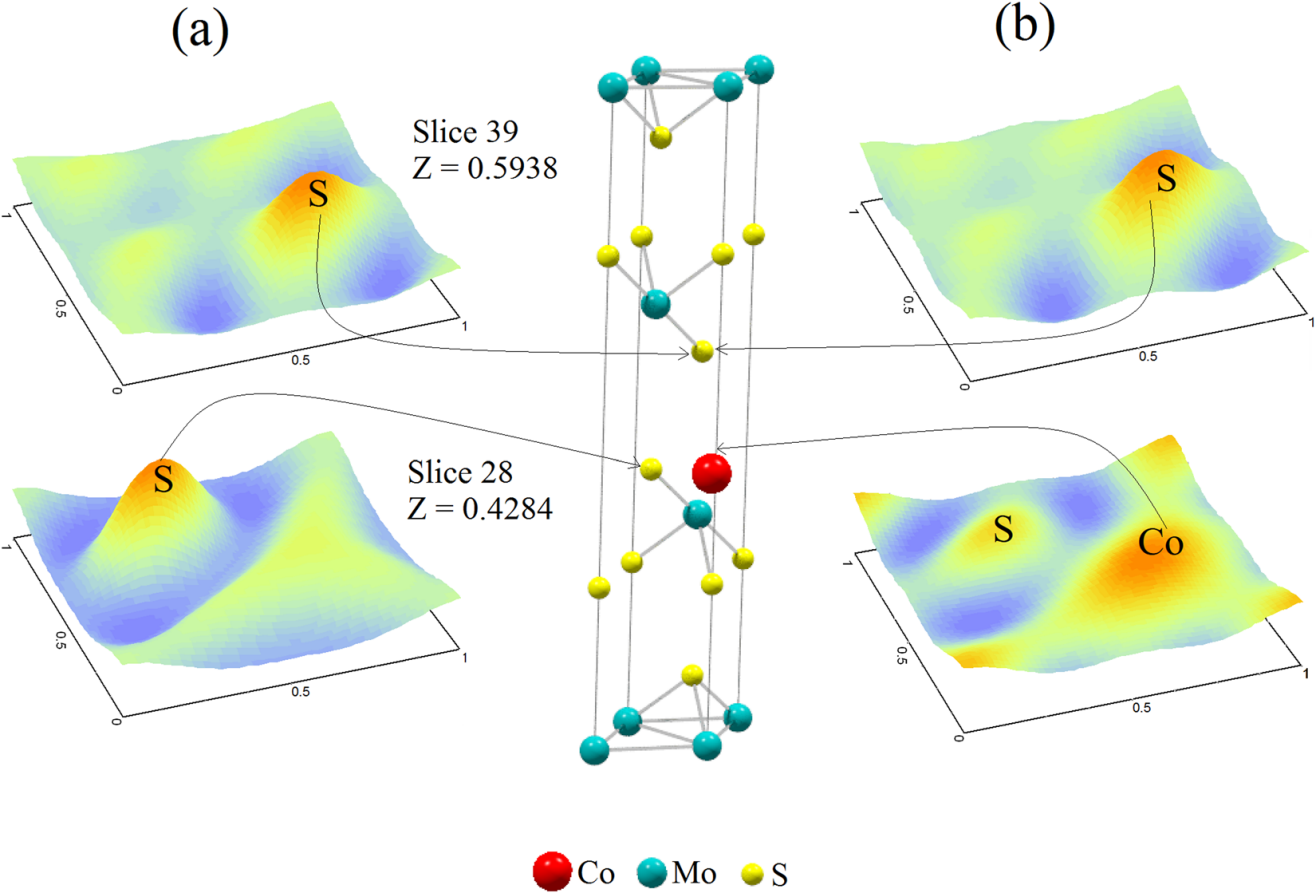
(**a**) Electron density maps to indicate cobalt atoms at the MoS_2_ crystallographic lattice and (**b**) for Cobalt atoms into the MoS_2_ lattice. (Data calculated using information from powder X-ray diffraction and DFT numerical simulations).

**Figure 4:**
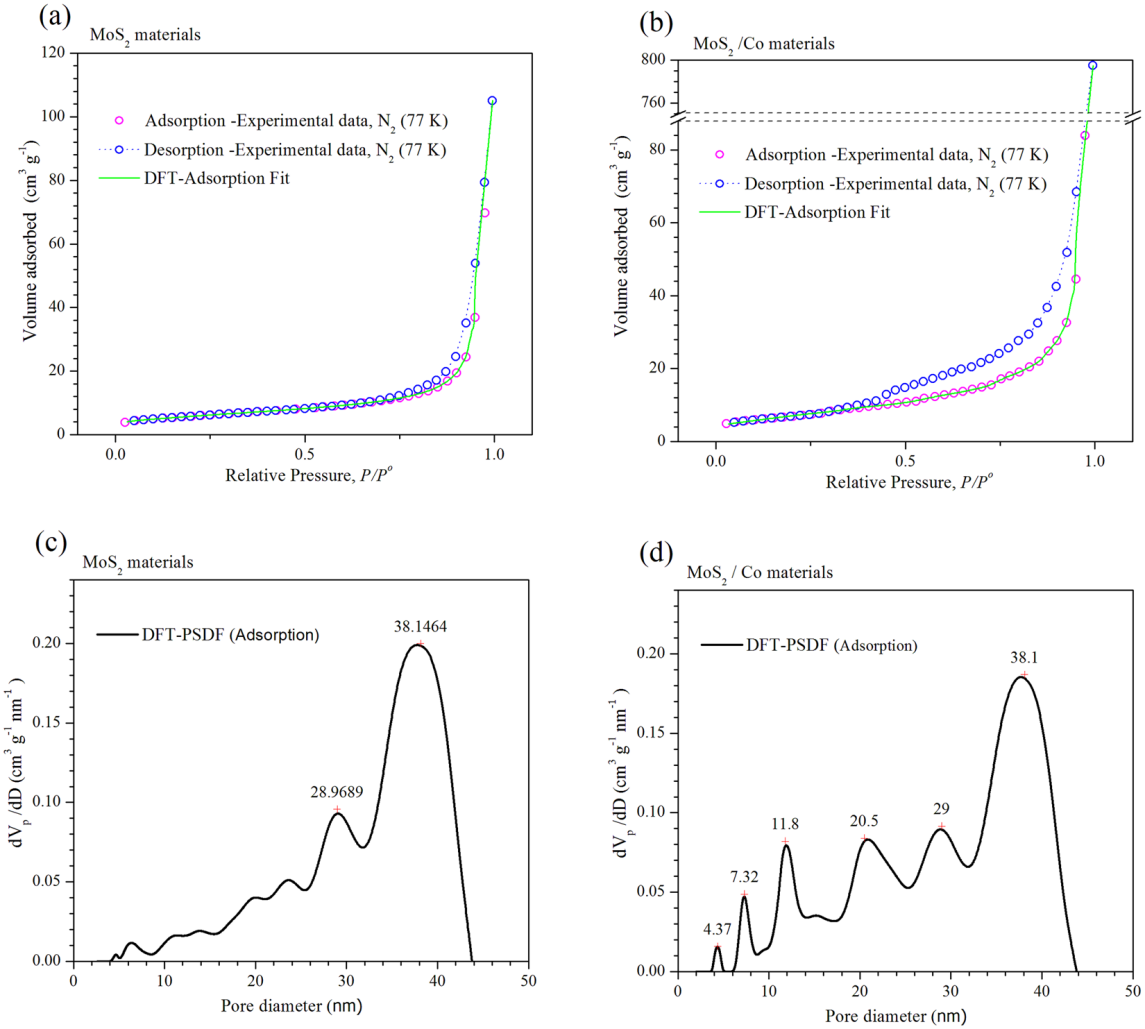
(**a** and **b**) Nitrogen adsorption/desorption isotherms run at 77 K for both cases MoS_2_ and Co/MoS_2_. (**c** and **d**) Numerical simulation of pore diameter distribution for MoS_2_ samples and (**d**) Co/MoS_2_ samples.

**Figure 5:**
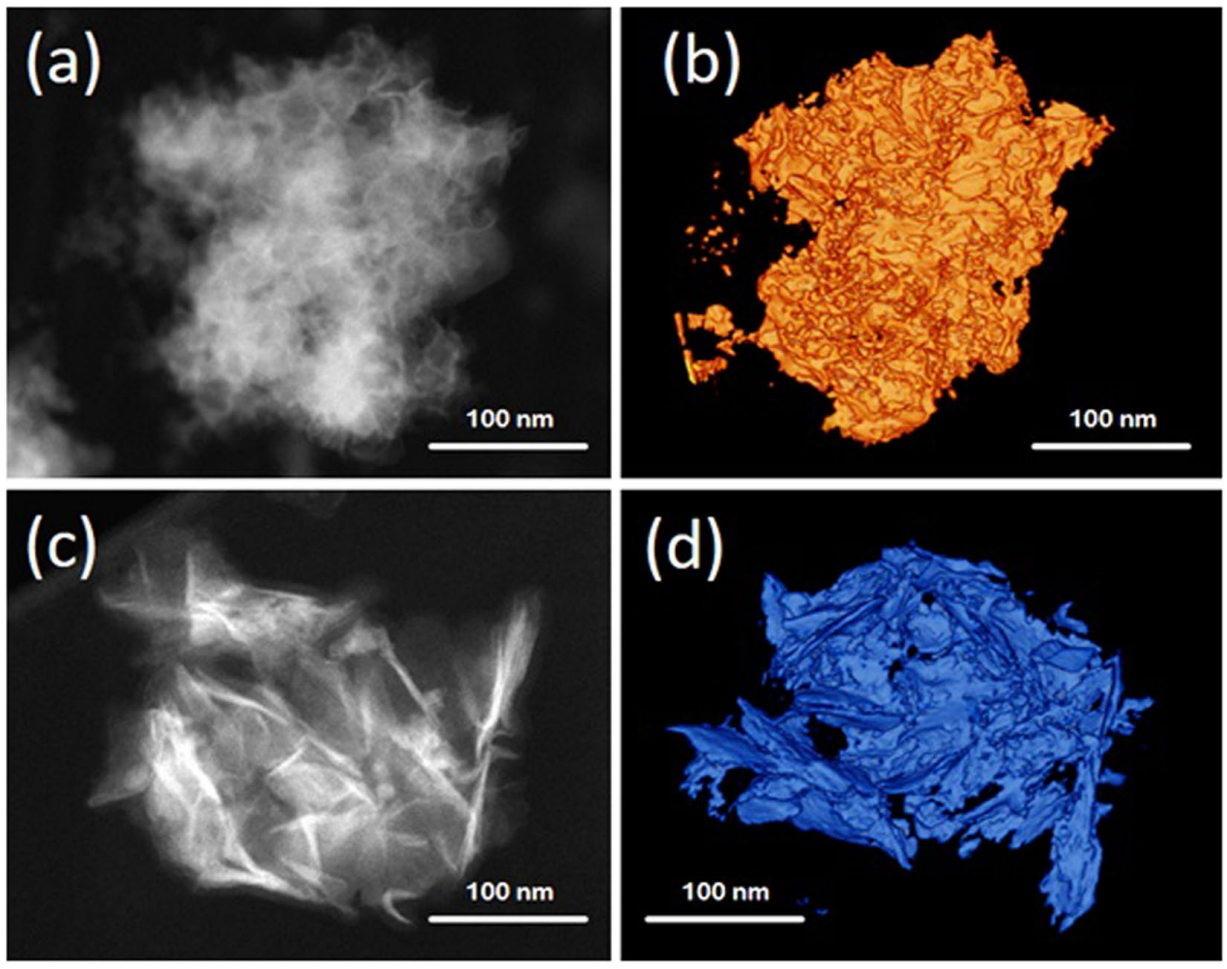
Scanning Transmission Electron Tomography for both MoS_2_ and Co/MoS_2_ samples. It is possible to determine surface dispersion and change of pore volume when cobalt atoms are present on the sample. Inset: High resolution image of specific section on both cases, one can observe large laminates growth due to addition of cobalt atoms into the MoS_2_ lattice.

**Figure 6:**
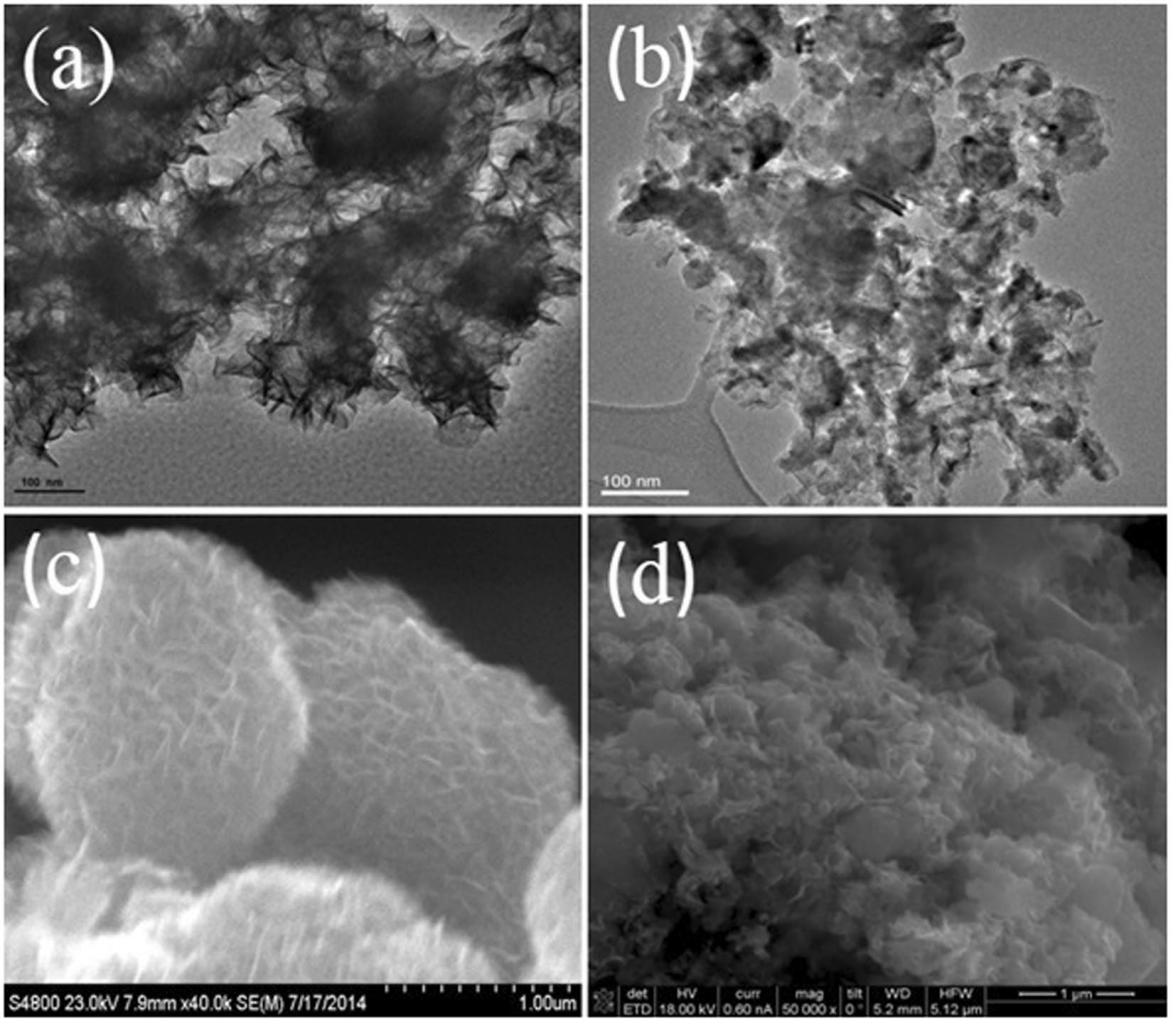
High resolution transmission electron microscopy images: (**a** and **b**) are TEM-images (scale bar 100 nm) corresponding to MoS_2_ and Co/MoS_2_ respectively. (**c** and **d**) are scanning electron microscopy images (scale bar 1.0 µm) of MoS_2_ and Co MoS_2_ using both techniques, to determine surface dispersion when cobalt atoms are added, as reported before^18^.

The crystallographic features of hydrothermally as-synthesized Cobalt promoted MoS_2_ catalysts were determined by X-ray powder diffraction (Ta﻿ble 1); this comprised the use of Rietveld’s refinement method to verify the MoS_2_ rhombohedral (R3m) symmetry, in agreement with recent work from Wang *et al*.^31^, from which a comparative analysis was performed as shown by Fig. 1, where one observes that a crystallographic variation occurs following cobalt addition during synthesis (Table﻿ 2), which can be explained in terms of Frenkel´s point defects^32^ (Table 3) caused by interstitial occluded cobalt atoms into the laminar MoS_2_ structure, as described by Lauritsen *et al*.^14^. This explains the expansion of about 12.9% (from 0.7500 to 0.8465) of c-axis in the primitive lattice, as demonstrated by Radial Distribution Function results, which indicates interatomic distance variations of 11.7% for S····Mo and 24.5% for S—Mo—S, with respect to S···Mo···S distance of 3.56 Å (Table﻿ 4); these are consistent with displacements of Mo atoms to interstitial sites as confirmed on electron density maps in Figs 2a,b and 3a,b with face *ab* normalized to 1 and with *c* = 0.42 (slice 28) and *c* = 0.59 (slice 39); some specific data are reported in Tables 1-3). In fact, an increase of 10.4% (from 0.16667 to 0.18400) in occupancy position of sulfur *O*_*S*(2)_ at the MoS_2_/Co lattice implies a stoichiometry variation for MoS_2_ to MoS_(2+y)_, thus leading to a molecular increase of crystal density of about 2.14% (from 4.989 to 5.096 g/cm^3^) as reported in Table 1); also, the mean crystallites size of MoS_2_ vary from 2 nm to 8 nm when cobalt is inserted, as discussed before^18^ and indicated in Table 2. The nitrogen adsorption profile of both MoS_2_ and MoS_2_/Co is consistent with type III isotherms, as shown in Fig. 4a,b with a pore size distribution with maxima at 29 and 38 nm for MoS_2_ while these figures vary from 4 nm to 38 nm for MoS_2_/Co as presented in Fig. 4c,d, thus indicating weak interactions between adsorbate and absorbent; also, a H3 type hysteresis loop (IUPAC standards) is observed in both cases, at the relative pressure (P/P°) interval between 0.4 and 0.45, thus leading to a model akin with slit-shaped pores, which was verified by electron tomography, as presented in Fig. 5a,b, thus indicating a higher N_2_ uptake after comparing MoS_2_ and MoS_2_/Co (i.e., a variation of more than 727%); the adsorption isotherm at *P/P°*∼ 0.8 suggests the formation of condensate at the pores neck for *P/P°* > 0.9, i.e., a liquid–vapor menisci move towards the cavity of the particles, until pores filling with condensates occurs. With the purpose of understanding better this phenomena a series of DFT numerical simulations were completed to determine relevant pore structure parameters (i.e., *S* ^*BET*^ */S* ^*DFT*^, *V*_*p*_^*DFT*^ and *Φ* ^*DFT*^)as shown in Table 5 ; We propose this parameter as an important one for gauging catalytic behavior of Co-promoted-MoS_2_ catalysts and as a complement of “electronic effects” induced by insertion of the Co promoter, because it modifies both textural and structural properties of MoS_2_; this is the first time that fractal dimensionality is shown as a result of Co-promoter insertion in spherical particles of MoS_2_ and the profound effects on both texture and structure as verified in the case of MoS_2_/Co system^32^. Thus, a summary of these results is presented in Table 5 where one observes an increase of 29% in *S* ^*BET*^ */S* ^*DF*^, with a further increase up to 110% for *V*_*p*_^*DFT*^ as a result of the overall increase of N_2_ uptake upon insertion of cobalt atoms in the MoS_2_ lattice. The calculation of Fractal dimension from transmission electron micrographs and the use of Neimark-Kiselev equation in ***D***_***s(****adsorption/desorption)*_ led to determine a decrease of 12% after insertion of cobalt (i.e., details are shown in Tables 6–7), thus implying a lower fractal dimension as exhibited in Fig. 5c,d for *r*_*k*_ values within the interval 6.05–40.44 nm for the MoS_2_ spherically shaped particles while *r*_*k*_ values fall within the interval 4.95–20.08 nm for MoS_2_/Co system that is spread on the surface, which is observed by STEM tomography (Table 7) as presented in Fig. 6a,b, from which the fractal dimension is obtained using *D*_*P*_, *D*_*B*_ *, D*_*I*_ and *D*_*M*_ methods, i.e., 1.17 and 1.05 are the fractal dimensions for MoS_2_ and MoS_2_/Co, while *D*_*B*_ decreases from 1.71 to 1.57due to the surface topology associated with cobalt addition; *D*_*I*_ indicates dispersion of the surface due to the cobalt presence and finally *D*_*M*_ determines a dense surface on MoS_2_/Co as observed by larger array of laminates revealed by high-resolution STEM (insets of Fig. 6a,b). Additionally, a second run of fractal calculations were done using TEM-2D images, from which fractal values show a decreasing trend with the cobalt presence, with values of *D*_*P*_ (25.0%), *D*_*B*_ (5.3%), *D*_*I*_ (17.0%) and *D*_*M*_ (16.0%) as well as when using scanning electron images with values of *D*_*P*_ (34.7%), *D*_*B*_ (28.0%), *D*_*I*_ (23.4%) and *D*_*M*_ (1.9%). See T﻿able﻿s 8–9.

**Table 1:** Lattice parameters obtained by Rietveld refinement from x-ray pow﻿der diffraction for pure spherical shaped MoS_2_ .

MoS_2_ R 3 m H (160)			Lattice: Rhombohedral			Occupancy
			Atomic fractional coordinates			
Atom	Type	Site	x	y	z	
Mo (1)	Mo^4+^	3a	0.0000	0.0000	1.0000	0.16667
S (1)	S^2−^	3a	0.0000	0.0000	0.2500	0.16667
S (2)	S^2−^	3a	0.0000	0.0000	0.7500	0.16667
**Lattice parameters (Å)**			**Angles**			
a	b	c	α	β	γ	Density (g cm^−3^)
3.1710	3.1710	18.3445	90°	90°	120°	4.992

**Table 2:** Lattice parameters obtained by Rietveld refinement from x-ray powder diffraction for cobalt promoted MoS_2_ .

Material	Molybdenite crystallite size (nm)	Density (g cm^−3^)	Atomic fractional coordinates of S (2)			*O*_*S* (2)_
			x	y	z	
MoS_2_	2 (0.4)	4.989	0.0000	0.0000	0.7500	0.16667
MoS_2_/Co	8 (0.8)	5.096	0.0000	0.0000	0.8465	0.18400

The number in parenthesis corresponds to the standard deviation.

*O*_*S* (2)_: *S* (2) occupancy (see Table 1).

**Table 3:** Statistical parameters after Rietveld refinement for both cases.

Material	*R*_*p*_	*R*_*wp*_	*χ* ^2^	*R*_*exp*_
MoS_2_	2.95	4.67	2.52	2.94
MoS_2_/Co	1.30	1.74	2.07	1.21

**Table 4:** Radial distribution function peaks values of MoS_2_ and Co/MoS_2_ samples.

Material	Interatomic distances r (Å)				
	Mo—Mo	S····Mo····S	S—Mo—S	S····Mo	Mo····Mo
MoS_2_^*^	3.171	3.563	4.770	5.575	6.383
MoS_2_^#^	3.170	3.560	4.720	5.710	6.360
MoS_2_/Co^#^	3.140			5.110	6.430

*Theoretical interatomic distances.

^#^Obtained by XRD.

—Bond, ----Only distance.

## Conclusion

The abovementioned results led us to conclude that chemical state and geometric features together might play an important role, i.e., cobalt atom radius is about 20% larger than sulfur atoms while cobalt ions (Co^+^) are about 40% the size of sulfur ion S^2−^; also, the specific hydrothermal synthesis method could contribute too. It was found that Co tends to occupy MoS_2_ edges, as determined by Rietveld’s refinement method, thus the “CoMoS phase” should be formed. Moreover, the use of Neimark-Kiselev set of equations led us to conclude that cobalt insertion into the MoS_2_ crystalline arrays induce a pore volume increase from 10 to 110 cm^3^/g, which provokes an increase of the diameter of cavities as well as formation of throats with diameters smaller than 29 nm and *r*_*k*_ < 14.4 nm, which explains the higher N_2_ consumption during the isotherm runs. Additionally, cobalt insertion promotes formation of large needle-like laminates with a stacking average of ~20 2D layers, as observed by high resolution STEM, with *D*_*s*_ values falling in a region where radii of curvature are smaller (14.4 nm), which means that smoother surfaces are formed inside the cavities. Also, a decreasing trend of *D*_*P*_, *D*_*B*_, *D*_*I*_ and *D*_*M*_ was found with insertion of cobalt in the MoS_2_ laminates (Table 4b–d), which is interpreted as enhancing the crystallite edge smoothing and surface density. This fundamental approach allowed to understand better the behavior of “CoMoS” phase type catalysts. Further work using a similar approach for “spent” MoS_2_/Co and MoS_2_ catalytic phases is underway for studying the role of carbon in the catalytic properties of those phases.

**Table 5:** Specific surface area, pore volume and mean pore size for both cases.

Material	*S* ^BET^ / *S* ^DFT^ (m^2^g^−1^)	*V*_P_^DFT^ (mm^3^ g^−1^)	*Φ* ^DFT^ (nm)
MoS_2_	20/21	10	29/38
MoS_2_/Co	26/27	110	4/7/12/21/29/38

Values were obtained from numerical DFT simulations and experimental isotherms.

**Table 6:** Fractal dimension parameters as calculated by Neimark-Kiselev from isotherm curves for MoS_2_ and Co/MoS_2_ samples.

Method of analysis	Materials	
	MoS_2_	MoS_2_/Co
*D*_*S (Adsorption)*_	2.47 (6.05–40.44)^#^	2.18 (4.95–20.08)^#^
*D*_*S (Desorption)*_	2.52 (6.05–49.40)^#^	2.25 (6.05–16.44)^#^

*Ds* scaling interval ∈ [2, 3]; ^#^Radius of curvature (nm).

**Table 7:** Fractal dimension parameters and porosity as calculated by different methods from STEM images.

Material	Method of analysis				
	*D*_*p*_	*D*_*B*_	*D*_*I*_	*D*_*M*_	STEM-porosity (%)
MoS_2_	1.17 ± 0.01	1.71 ± 0.02	1.83 ± 0.01	1.86 ± 0.01	14.6
MoS_2_/Co	1.05 ± 0.01	1.57 ± 0.01	1.64 ± 0.01	2.21 ± 0.01	23.6

*D*_*P*_, *D*_*B*_, *D*_*I*_ and *D*_*M*_ are measured from STEM-images; *D*_*p*_ scaling interval ∈ [1, 2]; *D*_*B*_ scaling interval ∈ [1, 2]; *D*_*I*_ scaling interval ∈ [1, 2]; *D*_*M*_ ≥ 1.

**Table 8:** Fractal dimension parameters as calculated by different methods from high-resolution TEM images for MoS_2_ and Co/MoS_2_.

Method of analysis	TEM images scale bar at 100 nm	
	MoS_2_	MoS_2_ /Co
*D*_*p*_	1.67 ± 0.25	1.25 ± 0.24
*D*_*B*_	1.69 ± 0.08	1.60 ± 0.06
*D*_*I*_	1.68 ± 0.37	1.40 ± 0.02
*D*_*M*_	2.00 ± 0.09	2.32 ± 0.17

*D*_*P*_, *D*_*B*_, *D*_*I*_ and *D*_*M*_ are measured from TEM-images; *D*_*p*_ scaling interval ∈ [1, 2]; *D*_*B*_ scaling interval ∈ [1, 2]; *D*_*I*_ scaling interval ∈ [1, 2]; *D*_*M*_ ≥ 1.

**Table 9:** Fractal dimension parameters as calculated by different methods from SEM images for both MoS_2_ and cobalt promoted MoS_2_ samples.

Method of analysis	SEM-images at 1.0 µm	
	MoS_2_	MoS_2_ /Co
*D*_*p*_	1.64 ± 0.03	1.07 ± 0.02
*D*_*B*_	1.90 ± 0.01	1.37 ± 0.01
*D*_*I*_	1.92 ± 0.01	1.47 ± 0.01
*D*_*M*_	1.98 ± 0.01	2.02 ± 0.02

*D*_*P*_, *D*_*B*_, *D*_*I*_ and *D*_*M*_ are measured from TEM-images; *D*_*p*_ scaling interval ∈ [1, 2]; *D*_*B*_ scaling interval ∈ [1, 2]; *D*_*I*_ scaling interval ∈ [1, 2]; *D*_*M*_ ≥ 1.

## Experimental Methods

### Catalyst Preparation

A black powder precipitate was synthesized by mixing 3 mmol of sodium molybdate (Na_2_MoO_4_.2H_2_O) and 9 mmol of thioacetamide (CH_3_CSNH_2_) when dissolved in 30 mL of deionized water, and then 0.5 g of sodium silicate (Na_2_SiO_3_.9H_2_O) was added into the solution under violent stirring. The pH value of the solution was adjusted to 6.0 by dropping 12 M hydrochloric acid (HCl) solution. 0.50 g of cobalt chloride (CoCl_2_) was added to the solution before the hydrothermal reaction at a precise temperature, i.e., 220 °C [inside reactor chamber] for 4 h, thus allowing a natural cooldown. The catalyst was washed using a 1 M of Sodium Hydroxide (NaOH) to remove possible residues, mainly silicic acid and this was dried at 200 °C in autoclave. The synthesis follows the stoichiometry reaction: **1)** 6CoCl_2_6H_2_O + 12Na_2_MoO_4_ + Na_2_SiO_3_ + 26HCl → H_4_SiCo_6_Mo_12_O_40_ + 26NaCl + 47H_2_O + 6Cl_2_. **2)** CH_3_CSNH_2_ + 2H_2_O → CH_3_COOH + NH_3 + _H_2_S. **3)** H_4_SiCo_6_Mo_12_O_40_ + 27H_2_S → 12Co_0:5_MoS_2_ + H_2_SiO_3_ + 3H_2_SO_4_ + 25H_2_O (for more information see Ramos *et al*.^18^).

### Powder XRD and Rietveld refinement

All diffraction patterns were obtained with a Bruker Advance D-8 diffractometer fitted with Bragg-Brentano geometry, using CuKα radiation and a Lynxeye type detector. The intensities were obtained in the 2-theta ranges between 10 and 100° with a step of 0.019447° and a measuring time of 10 s per point. The crystalline structures were refined by Rietveld’s method using TOPAS-Academic software^33^. All theoretical crystal density was calculated by the following equation: *ρ*_*crystal*_ = (Z)(MM)(Avogadro’s number)^−1^/Cell volume; where Z is the number of molecules per cell and MM is the molecular weight. Radial Distribution: The distance (r) between atoms in MoS_2_ and MoS_2_(Co) crystallites was obtained by Radial Distribution Function G(r) up to 6.5 Å, in a Siemens D500 diffractometer fitted with a molybdenum anode X-ray tube. The intensities were measured in a step-by-step mode of 0.01°, from 2 to 110° (2θ), using the Radiale program^34^.

### Adsorption/Desorption isotherms

All the measurements of N_2_ sorption isotherms were obtained at 76 K using a Quantachrome Autosorb 3B instrument under N_2_ and He gases (UHP grade) conditions. Prior to isotherms runs, all the samples were outgassed for 12 h at 473 K. The specific surface areas were calculated from desorption isotherms using BET equation, while the pore structure parameters were determined by non-local density functional theory.

### Experimental HRTEM microanalysis and Electron Tomography

Imaging of ultra-high resolution TEM was performed in an FEI Tecnai F20 instrument operating at 200 kV in STEM mode. Images were acquired every 2°, from −70 to + 70, totaling 71 images for each tilt series. The HRTEM images were collected in a JEOL ARM (200 F) instrument at operational voltage of 200 kV, which was fitted with a Cs corrector (CEOS GmbH) and FEG-STEM/TEM unit; a HAADF probe size was set to 0.095 nm with a current of 23.2 pA for bright field imaging, the condenser lens aperture size was set to 40 µm. A camera length (CL) of 8 cm/6 cm and collection angle of 68–280 mrad/90–270 mrad was set for STEM images, to eliminate contributions from un-scattered beams. The specimens were prepared for electron microscopy by crushing the powder between two glass slides, and rubbing a holey carbon grid across the crushed powder to capture the material. All images were reconstructed using Gatan Digital Micrograph® computational software and these were aligned with respect to each other using the center of mass of the particle^35^. These were reconstructed using total variation (TV) regularization, and visualized using the tomviz software^36^.

### Computational Software

Novawin 11.03 package was used for all the numerical simulations for porous size distribution and textural properties.

## Electronic supplementary material

Supplemental Material
