# Supplementary material for: Electron tomography and fractal aspects of MoS2 and MoS2/Co spheres
Source: Sci Rep. 2017 Sep 26;7:12322. doi: 10.1038/s41598-017-12029-8 (PMC5615070; doi:10.1038/s41598-017-12029-8)
Supplement: Supplementary file 1 — Supplemental Material [file 41598_2017_12029_MOESM1_ESM.pdf]

Electron tomography and fractal aspects of MoS_2 and MoS_2/Co spheres

Manuel Ramos[b], Félix Galindo-Hernández[a], Ikle Arslan[c], Toby Sanders[c], José Manuel Domínguez[a]

[a] *Instituto Mexicano del Petróleo (IMP). Eje Central Lázaro Cárdenas Norte 152 Col. San Bartolo Atepehuacan, México D.F. C.P 07730.*
[b] *Departamento de Física y Matemáticas, UACJ-Instituto de Ingeniería y Tecnología, #450 Avenida del Charro, Cuidad Juárez, 32310, México.*
[c] *School of Mathematical and Statistical Sciences, Arizona State University, Tempe, AZ, 85281, United States*
[d] *Physical Sciences Division, Pacific Northwest National Laboratory, 902 Battelle Blvd., Richland, Washington 99354, United States*

SUPLEMENTAL MATERIAL

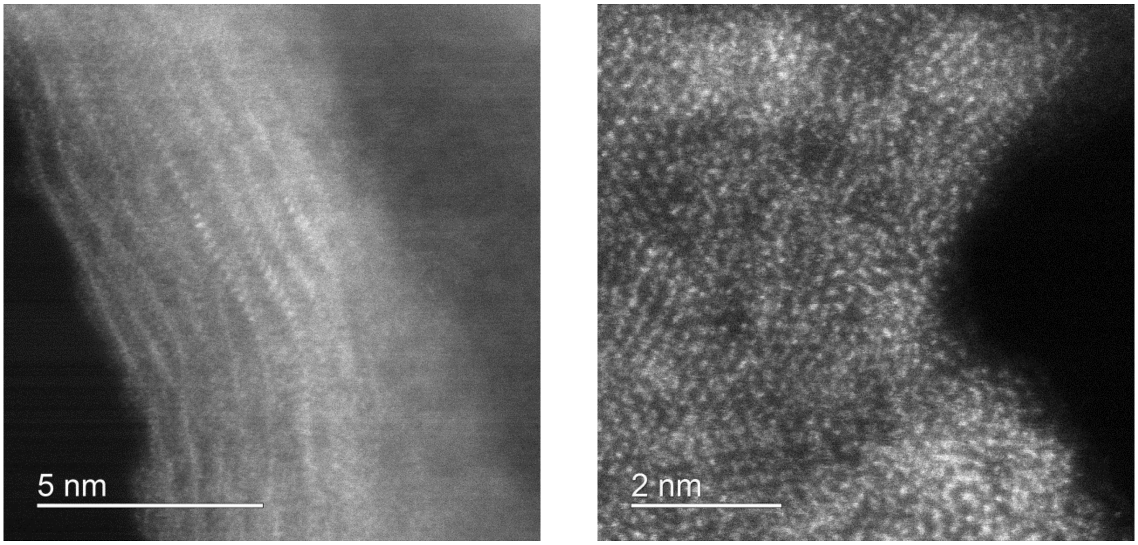

Extended Data Figure 1 │ High-resolution scanning transmission electron micrographs of MoS_2 samples. Left image it is possible to observed laminar structure with some bending curvatures. Right image presents a turbostratic arrangement of (001)-bassal plane, we can compare with results as presented by Garcia, A. *et al.*, "Analysis of electron beam damage of exfoliated MoS_2 sheets and quantitative HAADF-STEM imaging", Ultramicroscopy,**146** 33–38, (2014).

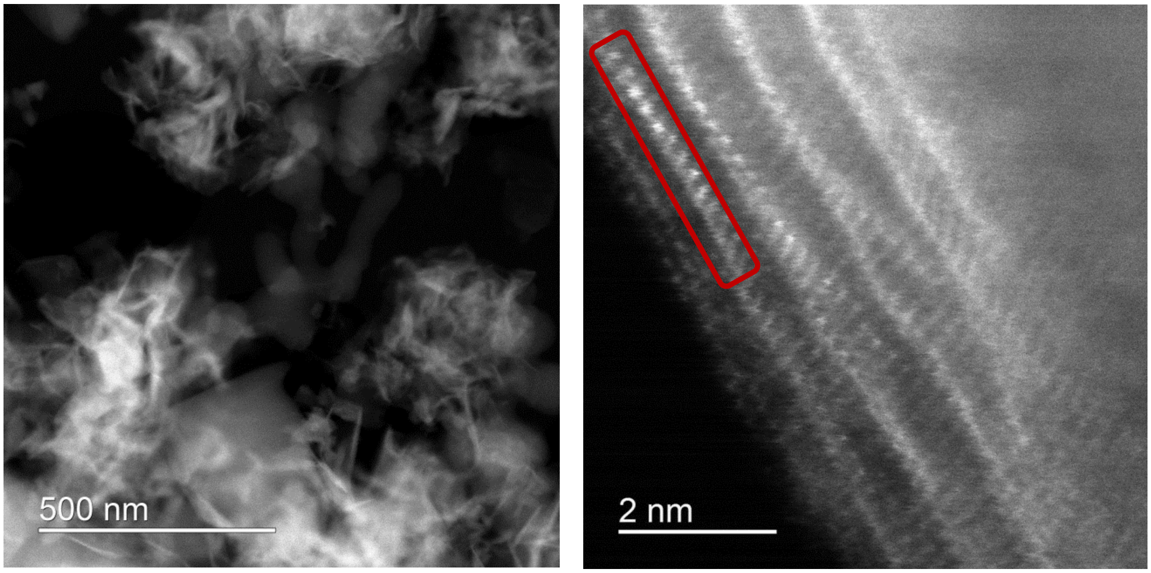

Extended Data Figure 2 | High-resolution scanning transmission electron micrographs of Co/MoS_2 samples. Left image it is possible to observed laminar neddle-like structures dispersed. Right image presents layered arrangement of with bright spots corresponding to cobalt atoms (***Z***-contrast) as compared with results presented by Garcia, A. *et al.*, "Analysis of electron beam damage of exfoliated MoS_2 sheets and quantitative HAADF-STEM imaging", Ultramicroscopy,**146** 33–38, (2014).

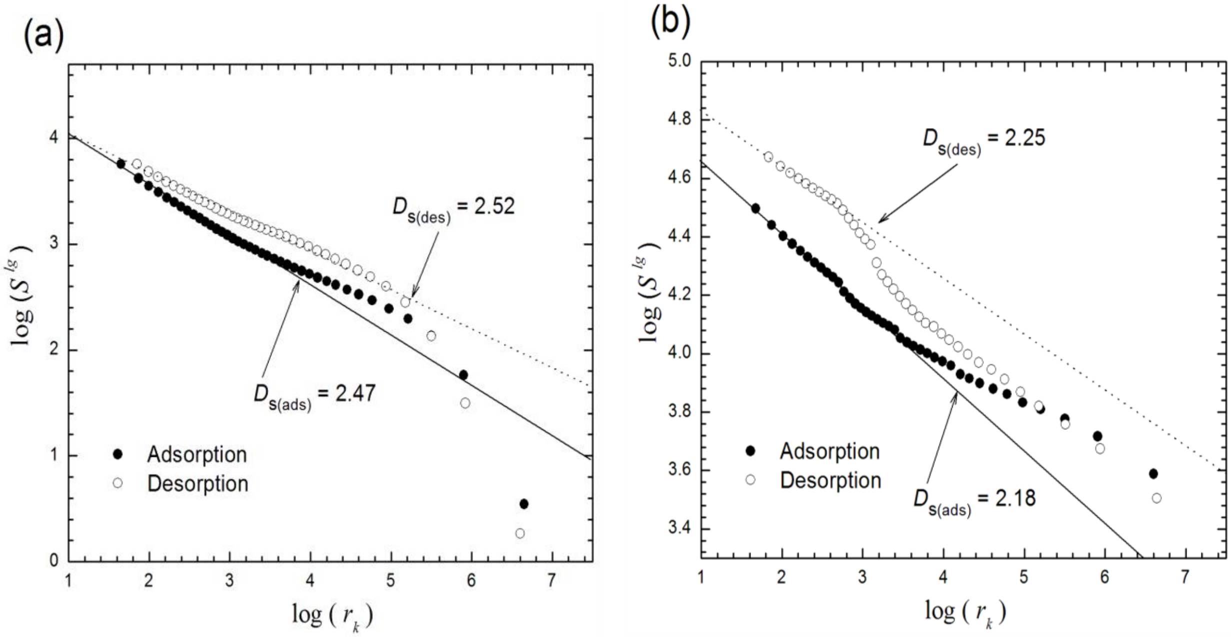

Extended Data Figure 3 | Neimark-Kiselev fractal analysis of the adsorption-desorption isotherms. (a) MoS_2 samples and (b) MoS_2/Co samples.

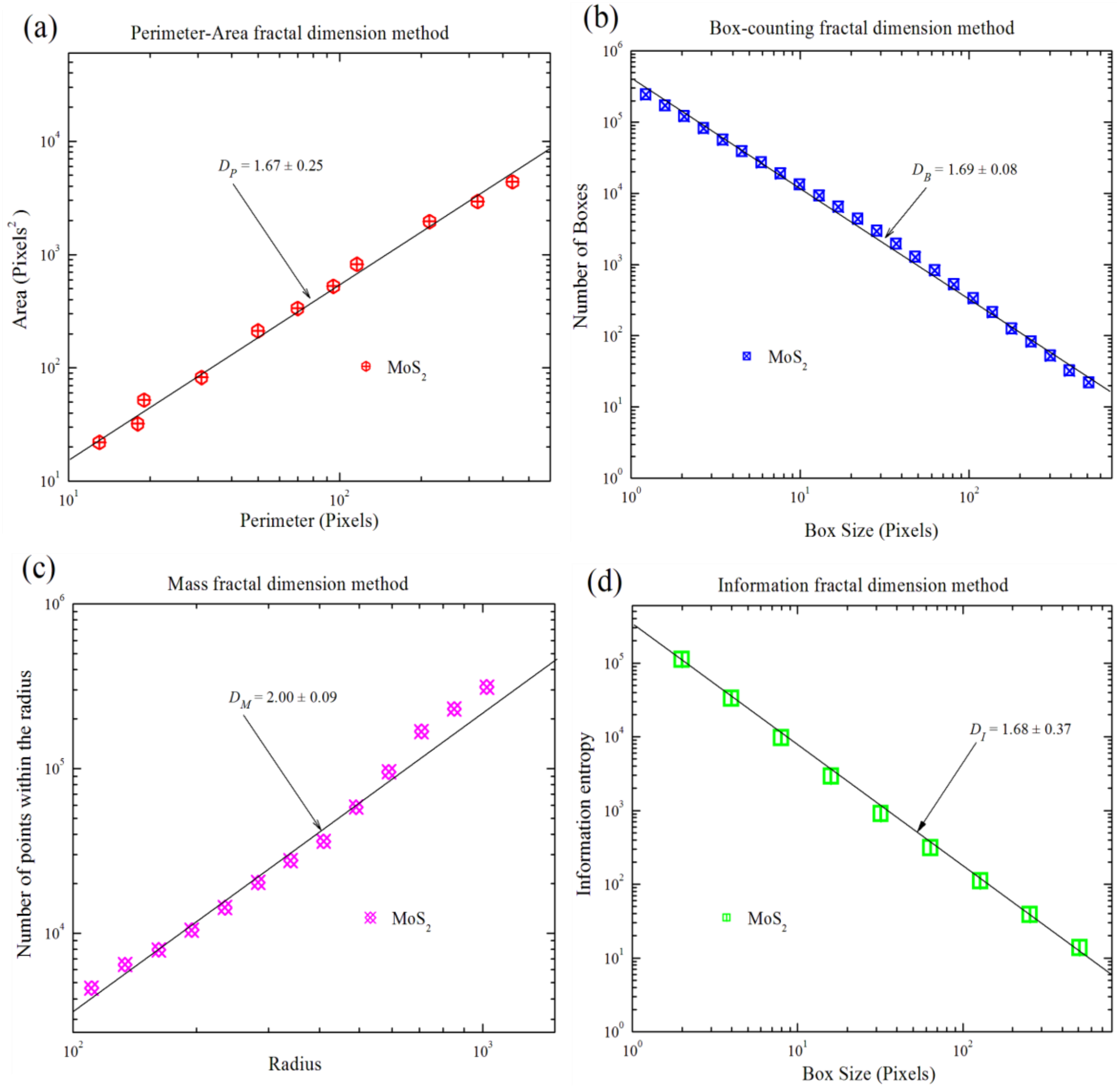

Extended Data Figure 4 | Representative plots performed on TEM of MoS_2 electron micrographs [Figure 6]. (a) Perimeter-area, (b) Box-counting, (c) Mass and (d) Information fractal dimension methods.

MATHEMATICAL APPROACH

The Density Functional Theory provide not only a microscopic model of adsorption but also a more realistic description of the thermodynamic properties related to pore fluid, which bridge the gap between molecular and macroscopic level. Using this particular method it is possible to determine local fluid structure near curved solid walls and gives us the theoretical adsorption–desorption isotherms in porous structure.

The local fluid density ρ of adsorbate confined in a pore at given chemical potential μ and temperature T is determined by minimization of the grand thermodynamic potential Ω:

$$\Omega = \int \rho[f(r,\rho,\bar{\rho}) + u^{ext}(r) - \mu]dr$$

where $\rho, \bar{\rho}$ are the local and smoothed fluid densities; $f(r,\rho,\bar{\rho})$ is the molar Helmholtz free energy; $u^{ext}(r)$ is external potential exerted by the solid; μ is the chemical potential. The molar Helmholtz free energy is split into the ideal term $kT[Ln(\Lambda^3\rho) - 1]$, excess term $f_{exc}(\bar{\rho})$ due to repulsion, and the intermolecular interaction potential $u(r)$ due to attraction:

$$f(r,\rho,\bar{\rho}) = kT[Ln(\Lambda^3\rho) - 1] + f_{exc}(\bar{\rho}) + u(r)$$

Here k is the Boltzmann constant and Λ is the de Broglie wavelength. The smoothed density $\bar{\rho}$ is a weighted average:

$$\bar{\rho}(r) = \int \rho\,(r')\omega\big(|r - r'|, \bar{\rho}(r)\big)dr'$$

where $\omega\big(|r - r'|, \bar{\rho}(r)\big)$ is the weighting function.

Neimark-Kiselev fractal analysis: The fractal dimension is a measure to classify and quantitatively compare complex chaotic patterns such as surfaces of the porous materials. After the DFT treatment, Neimark-Kiselev method can be used to obtain the fractal dimension values. Combining thermodynamic and fractal arguments:

$$S_{lg} = K(r_k)^{2-D}$$

Where D is the surface fractal dimension, K is a constant, r_k is the mean radius of curvature of the adsorbate-vapor interface, given by the Kelvin equation,

$$r_k = \frac{2\gamma V_m^{DFT}}{RTln\left(\frac{P^0}{P}\right)}$$

And S_{lg} is the adsorbate-vapor interface area, given by the Kiselev equation,

$$S_{lg} = \frac{RT}{\gamma} \int_{n}^{n_{max}} ln\left(\frac{P^0}{P}\right) dn$$

with R being the universal gas constant, T the adsorption temperature, γ the adsorbate surface tension, V_m the adsorbate molar volume, and n and n_{max} the amounts of gas adsorbed at given P/P^0.

In our case of study for low dimension surface topology of Co/MoS_2 spheres, the fractal dimension values indicate topographic changes onto Co/MoS_2 surface´s which may be related inclusion of Co atoms at laminar sites; this approach can be used as fundamental aspect to understand catalytic performance and the selectivity [29-33].

[29] Samuel J., Ottolenghi M. & Avnir D. Diffusion Limited Reactions at Solid-Liquid Interfaces: Effects of Surface Geometry. *J. Phys. Chem.* **95**, 1890-1895 (1991).
[30] Avnir D. *et al.* Fractal Analysis of Size Effects and Surface Morphology Effects in Catalysis and Electrocatalysis. *Chaos* **1,** 397-410 (1991).
[31] Seri-Levy A. & Avnir D. Effects of Heterogeneous Surface Geometry on Adsorption. *Langmuir* **9,** 3067-3076 (1993).
[33] Neimark A.V. Calculating Surface Fractal Dimensions of Adsorbents *Ads. Sci. Tech.* **7**, 210-219 (1991).
